# Resting‐state brain entropy in right temporal lobe epilepsy and its relationship with alertness

**DOI:** 10.1002/brb3.1446

**Published:** 2019-10-12

**Authors:** Muhua Zhou, Wenyu Jiang, Dan Zhong, Jinou Zheng

**Affiliations:** ^1^ Department of Neurology The First Affiliated Hospital of Guangxi Medical University Nanning China

**Keywords:** alertness, brain entropy, resting‐state fMRI, temporal lobe epilepsy

## Abstract

**Background:**

To date, no functional MRI (fMRI) studies have focused on brain entropy in right temporal lobe epilepsy (rTLE) patients. Here, we characterized brain entropy (BEN) alterations in patients with rTLE using resting‐state functional MRI(rs‐fMRI) and explored the relationship between BEN and alertness.

**Method:**

Thirty‐one rTLE patients and 33 controls underwent MRI scanning to investigate differences in BEN and resting‐state functional connectivity (rs‐FC) in regions of interest (ROIs) between patients and controls. Correlation analyses were performed to examine relationships between the BEN of each ROI and alertness reaction times (RTs) in rTLE patients.

**Results:**

Compared with controls, the BEN of rTLE patients was significantly increased in the right middle temporal gyrus, inferior temporal gyrus, and other regions of the left hemisphere and significantly decreased in the right middle frontal gyrus and left supplementary motor area (*p* < .05). The rs‐FCs between the ROIs (at *p* < .01, with the left superior parietal lobule and right precentral gyrus defined as ROI1 and ROI2, respectively) and the whole brain showed an increasing trend in rTLE patients. In addition, the BEN of ROI2 was associated with the intrinsic alertness and phasic alertness RTs of patients with rTLE.

**Conclusions:**

Our findings suggest that BEN is altered in patients with rTLE and that decreased BEN in the right precentral gyrus is positively related to intrinsic and phasic alertness; the abnormal FC in the brain regions with altered entropy suggests a reconstruction of brain functional connectivity. These findings suggest that BEN mapping may provide a useful tool for probing brain mechanisms related to TLE.

## Graphical abstract

Decreased in BEN was associated with the intrinsic alertness RT and phasic alertness in patients with rTLE. The abnormal FC in brain regions with altered entropy suggests reconstruction of brain functional connectivity.

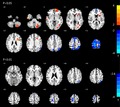

## 1 INTRODUCTION

Temporal lobe epilepsy (TLE) is one of the most common types of epilepsy. The root cause of epileptic seizures is the highly synchronized abnormal discharge of cerebral neurons. Epileptic seizures can lead to cognitive impairments in learning, memory, language, and intelligence (Hermann et al., 2006; Xu et al., 2018), which seriously affects the quality of life of epileptic patients.

MRI studies have revealed that brain functional connectivity, the amplitude of low‐frequency fluctuations (ALFFs), regional homogeneity (ReHo), the fractional anisotropy (FA) of white matter fibers, topological properties, and many other indicators are altered in patients with TLE (Bernhardt, Hong, Bernasconi, & Bernasconi, 2013; DeSalvo, Douw, Tanaka, Reinsberger, & Stufflebeam, 2014; Jiang, Li, Chen, Ye, & Zheng, 2017; Liao et al., 2010); thus, the function and structure of the brain in TLE patients are damaged to varying degrees. However, how the regularity and complexity of rs‐fMRI time series are altered in rTLE patients is still unknown.

Entropy is a nonlinear, dynamic parameter. The concept of entropy was proposed by the German physicist Clausius in 1865. Entropy can be used to measure quantities such as the incidence of new information in a time series and the randomness of a system (Shannon, 1948), which have important applications in cybernetics, probability theory, number theory, astrophysics, the life sciences, and other fields. An increase in entropy suggests an increase in the randomness of a system, indicating that the dynamic system activity is less predictable and less organized. The measures of entropy include approximate entropy (Pincus, 1991), sample entropy (Richman & Moorman, 2000), and so on. The human brain is a highly complex organ, and it must maintain stable entropy to retain its normal functions (Bergström, 1969). Entropy has long been used to evaluate different brain states through analysis of electrophysiological data, such as EEG data and heart rate variability (Burns & Rajan, 2015; Chen, Chen, Li, Wang, & Liu, 2016; Hogan et al., 2015; Lu et al., 2015). Li, Yan, Karmakar, and Liu (2015) found that the distribution of entropy was significantly higher during epileptic seizures than during the interictal period in patients with epilepsy and in healthy individuals. The entropy detected during the interictal period in epileptic individuals was also higher than that in normal people. In another study, Sridevi et al. (2019) recorded EEG data for 29 epileptic seizures in 18 TLE patients. This study showed that the spectral entropy of the T1, T2, T3, and T4 channels decreased significantly during epileptic seizures. This result suggests that entropy changed most in the epileptogenic area, as the T1, T2, T3, and T4 channels are located in the temporal lobe. Because of the frequently synchronous discharge of neurons in epilepsy, and because the entropy observed from the EEG changed most at the epileptogenic area, we suspect that the BEN determined from an rs‐fMRI time series will be increased in the brains of temporal epileptic patients, especially at the temporal lobe. In recent years, researchers have used entropy in fMRI time series analyses, offering a new perspective on the state of the brain (Araujo et al., 2003; Baumgartner, Somorjai, Summers, Richter, & Ryner, 2000; Wang, Li, Childress, & Detre, 2014). Sample entropy is well suited for short data sets such as fMRI data (Sokunbi, 2014). Entropy in the brain is variable among healthy individuals of different age (Smith, Yan, & Wang, 2014; Yao et al., 2013) and sex (Yao et al., 2013) and among individuals with different neurological diseases, such as multiple sclerosis (Zhou, Zhuang, et al., 2016), Alzheimer's disease (Liu et al., 2013; Niu et al., 2018; Wang et al., 2017), attention deficit hyperactivity disorder (ADHD) (Akdeniz, 2017; Sato, Takahashi, Hoexter, Massirer, & Fujita, 2013; Sokunbi et al., 2013), and schizophrenia (Sokunbi et al., 2014; Yang et al., 2015). The present study used an rs‐fMRI time series to calculate brain entropy as a parameter of complexity and regularity. In reports on this subject, there are disagreements regarding whether the complexity of the brain increases or decreases in disease and during aging. Vaillancourt and Newell (2002) suggest that increases or decreases in brain complexity in disease and aging not only depend on the internal dynamics of the brain but also on changes resulting from short‐term specific tasks or environmental demands. In general, the use of brain entropy as a measure of the complexity and regularity of the brain in fMRI analysis is a meaningful, novel way to understand the brain and brain diseases. The results of this study may improve the mechanistic understanding of impaired neural function in TLE.

The recurrent attacks experienced by TLE patients cause dysfunctions in learning, memory, language, intelligence, and other cognitive processes, which seriously affect their quality of life. Attention is the foundation of all cognitive functions. Posner (2008) suggested that attention can be divided into three networks: alertness, orientation, and execution control. The function of the alertness network is to achieve and maintain a state of high vigilance, which is the basis of continuous information processing. Depending on whether a warning cue is present before a target, alertness can be subdivided into phasic alertness and intrinsic alertness (Petersen & Posner, 2012; Sturm & Willmes, 2001). The former represents the short‐term ability to enhance response readiness following detection of an external cue, whereas the latter refers to the internal control of arousal or wakefulness in the absence of such a cue; both can be assessed by measuring reaction times. In our previous study, we found that patients with temporal lobe epilepsy have impaired vigilance functions (Chen et al., 2015; Zheng et al., 2012). In an attention network test (ANT) task‐related functional MRI analysis, we (Zheng et al., 2012) found significantly weaker activation of specific brain regions (including the cerebellum, right occipital lobe, right frontal lobe, and brainstem) in TLE patients than in healthy controls. In addition, our previous study showed that the functional connectivity (FC) between the thalamus and the anterior cingulate gyrus in patients with rTLE (Chen et al., 2015) was associated with vigilance, and the integrity of the resting‐state functional network was disrupted. Brain entropy is an index representing the complexity of the brain. In previous studies, it was found that brain entropy has a relationship with alertness. Mateos, Guevara Erra, Wennberg, and Perez Velazquez (2018) used EEG, intracranial EEG, and magnetoencephalography (MEG) recordings in subjects during different states of consciousness: resting wakefulness, different sleep stages, and epileptic seizures. They found that when people wake up, brain entropy increased, while it decreased in sleep. With increasing sleep depth, the entropy further decreased. Entropy increased during the interictal period and decreased during seizures, indicating that entropy changes with alert state. In other studies, entropy‐based EEG signals were negatively correlated with alertness (Bruzzo et al., 2008; Zhao et al., 2015), and permutation entropy was mainly distributed in the parietal and frontal lobes (Zhao et al., 2015). We know that there is a debate about whether the entropy in disease and the aging process increases or decreases, but alertness changes do affect entropy. However, there are no studies on the relationship between changes in brain entropy on fMRI and vigilance in rTLE.

To the best of our knowledge, no fMRI studies have focused on brain entropy in rTLE patients. In this study, we studied changes in brain entropy in patients with rTLE based on an rs‐fMRI time series. We hypothesized that irregularities in the rs‐fMRI time series with TLE would affect the FC. To test this hypothesis, we calculated the FCs of ROIs with altered BEN and compared patients with controls. We also used an ANT test to evaluate alertness functions in patients with rTLE, and relationships between the alertness RT and BEN of the ROIs in the patient group were assessed. We aimed to understand damage to brain functions in rTLE and the effect of abnormal brain dynamic system activity on alertness from a new perspective.

## 2 METHODS AND MATERIALS

### 2.1 Subjects

Thirty‐one rTLE patients [mean age ± standard deviation (*SD*) = 27.87 ± 7.10 years, mean years of education ± *SD *= 13.8 ± 3.1 years, 14 males] were recruited from the Epilepsy Clinic of the First Affiliated Hospital of Guangxi Medical University from September 2013 to February 2016. Epilepsy was diagnosed in accordance with the diagnostic criteria proposed by the International League Against Epilepsy (ILAE; Glauser et al., 2006). rTLE patients met the following criteria: (a) the presence of clinical symptoms, including seizures suggesting that the epileptogenic focus was located in the temporal lobe, as well as abnormal emotional symptoms (psychiatric symptoms and epigastric rising; automatisms; and dystonic posturing of the limbs); (b) MRI results showing right hippocampal sclerosis, atrophy, or aberrations in the right temporal lobe; and (c) an ictal or interictal electroencephalogram revealing epileptic discharges in the right temporal lobe (rTLE patients met criteria b, c, or both). All patients were regularly administered antiepileptic drugs in accordance with the ILAE treatment guidelines and had suffered no epileptic seizures within 24 hr. The exclusion criteria were as follows: (a) a diagnosis of rTLE in a patient with other serious bodily diseases; (b) a history of drug abuse; and (c) a Mini‐Mental State Examination score < 24.

Thirty‐three healthy controls [mean age ± *SD *= 27.69 ± 4.86 years, mean years of education ± *SD *= 13.9 ± 2.5 years, 16 males] were matched according to sex, age, and education level with the rTLE patients. All subjects underwent basic physical examinations of the nervous system (including visual acuity and visual field examination) in the first visit and before the experiment and showed no positive signs. All of the subjects were right‐handed and were informed in detail about the experiment. We had access to information that could identify individual participants during or after data collection. Written informed consent was obtained from all subjects. This study was approved by the Ethics Committee of the First Affiliated Hospital of Guangxi Medical University.

### 2.2 Neuropsychological evaluation using the ANT

**Figure 1:**
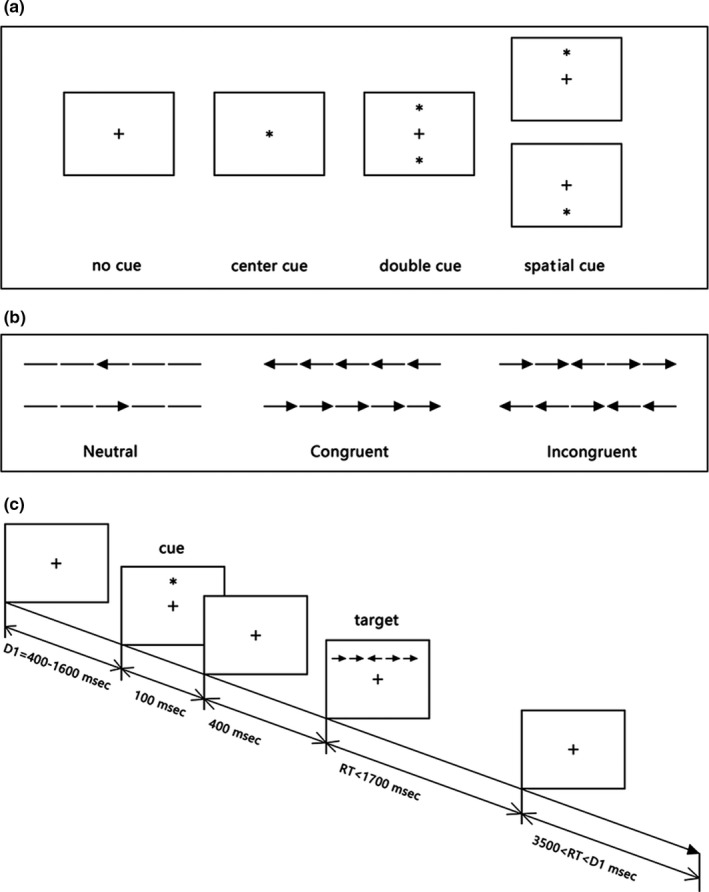
The procedure of the attention network test. (a) The four cue conditions; (b) the six stimuli used in the present experiment; (c) an example of the procedure

To evaluate alertness, the subjects were administered an ANT, which is a commonly administered neuropsychological test proposed by Fan, McCandliss, Sommer, Raz, and Posner (2002). In this test (Figure 1), a "+" in the center of a computer screen acts as a fixation point, a "*" represents the warning cue, and a "→" represents the target signal. The warning signal was presented in one of the following four ways: no cue; a cue appearing in the center position; cues appearing above and below the center position simultaneously (double cues); and a cue appearing only above or below the center position (space cue). In addition, the target signal was presented in one of two ways: the directions of the middle arrow and the other four arrows were the same (all arrows pointing away from the target signal), or only the middle arrow pointed to the target signal (and the other arrows pointed away from it). For this test, the subjects sat 60 cm away from the computer screen, placed their fingers on a reaction key, and were told to quickly push the reaction key according to the target signals. The computer recorded the subjects' correct and incorrect responses and RTs. The duration of the whole experiment was approximately 25 min. The total alertness network RT was equal to the intrinsic alertness RT (no cue) minus the phasic alertness RT (double cues). Differences in alertness were assessed using a two‐sample *t* test in SPSS (version SPSS 16, RRID:SCR_002865). *p* < .05 was considered significant.

### 2.3 MRI data acquisition

MRI scans were acquired using an Achieva 3 T MRI scanner (Philips) with a 12‐channel head coil. The subjects were instructed to lie still, close their eyes, and avoid falling asleep during the scan. Headphones and padding were used to reduce noise and prevent head motion. The scanning parameters were as follows: (a) a two‐dimensional structural MRI with a T2‐weighted sequence (repetition time (TR)/echo time (TE) = 3,000/80 ms, field of view (FOV) = 24 × 24 cm^2^, NEX2, thickness = 5 mm, and slice gap = 1 mm); (b) a high‐resolution anatomical scan using a three‐dimensional (3D) magnetization‐prepared rapid gradient echo (MPRAGE) sequence (TR/TE = 2,000/3.5 ms, matrix = 512 × 512 × 156, and FOV = 240 × 240 × 156 mm^3^); (c) an rs‐fMRI scan (TR/TE = 2,000/30 ms, FOV = 220 × 220 mm^2^, slice thickness = 5 mm, slice gap = 1 mm, acquisition matrix = 64 × 64, flip angle = 90°, and voxel size = 3.44 mm × 3.44 mm × 6.00 mm, with 31 slices and 180 dynamics); and (d) diffusion tensor imaging (DTI) with TR/TE = 1,000/15 ms; matrix = 128 × 128; FOV = 240 × 240 mm^2^; 64 axial slices (thickness = 2.0 mm); and 32 nonlinear gradient directions (*b* = 0, 1,000 s/mm^2^).

### 2.4 BEN analysis

The rs‐fMRI data were analyzed using DPARSFA V2.3 (http://rfmri.org/DPARSF_V2_3, RRID:SCR_002372), which is based on SPM8 (http://www.fil.ion.ucl.ac.uk/spm/software/spm8/, RRID:SCR_007037) and MATLAB 7.14.0 (MathWorks, Natick, MA, USA, RRID:SCR_001622), to correct for head movements. Participants with head motion of more than 2 mm of translation or 2° of rotation were excluded. Three patients were excluded based on these criteria. In total, 31 patients with rTLE and 33 healthy controls were included in the subsequent analysis. The Brain Entropy Mapping Toolbox (Wang et al., 2014, https://cfn.upenn.edu/~zewang/BENtbx.php, RRID:SCR_014470) of MATLAB was used to generate BEN maps. BEN was calculated at each voxel, consisting of the following steps: (a) image alignment; (b) segmentation of 3D T1 images; (c) slice timing and motion correction; (d) creation of a brain mask; (e) calculation of the tSNR and temporal image variance; (f) registration of the 3D T1 images to EPI images; (g) bandpass filtering (0.01–0.08 Hz); (h) smoothing (6‐mm full‐width at half‐maximum Gaussian kernel); (i) generation of BEN maps; (j) coregistration of the EPI images and BEN map to the 3D T1 images; (k) normalization using SPM8 segmentation; and (l) smoothing of the BEN maps. Before performing a group‐level analysis, Fisher's r‐to‐z transformation was applied to the BEN maps.

Statistical analysis of the BEN maps between the patients and controls was performed using the two‐sample *t* test in the Resting‐State fMRI Data Analysis Toolkit (REST, by Song Xiaowei, http://resting-fmri.sourceforge.net, RRID:SCR_009641). Age and sex were included as variables. The significance level was set at *p* < .05 and AlphaSim‐corrected (single voxel *p* < .01, cluster size ≥ 140). Significant clusters (*p* < .01) were defined as ROIs. In our study, the brain regions (significant at *p* < .05) that showed altered BEN in patients were distributed in various parts of the brain, while at *p* < .01, only two significant brain regions remained. Retaining all brain regions may result in more errors or deviations, while choosing more prominent brain regions reduces the probability of false positives. We then calculated the entropy values of the ROIs for each individual. Spearman's rank correlation analyses were performed using SPSS between the BEN values and the intrinsic alertness RT, phasic alertness RT, or alertness RT. Significance was set at *p* < .05. Entropy values have been reported to usually follow a non‐normal distribution (Li, Karmakar, Yan, Palaniswami, & Liu, 2016), so we performed a normality test analysis of the calculated entropy value (*p* = .07). Because the *p* value was close to .05 and our sample size was not large, we chose Spearman's rank correlation.

### 2.5 Seed‐based rs‐FC analysis of altered BEN regions

First, we preprocessed the rs‐fMRI data using DPARSFA V2.3 (http://rfmri.org/DPARSF_V2_3, RRID:SCR_002372). The first 10 dynamics were removed; slice timing and motion were corrected; 3D T1 images were registered to the EPI template in the Montreal Neurological Institute (MNI) space, with voxel size resampling of 3 mm × 3 mm × 3 mm; smoothing was performed (FWHM, 6‐mm); linear detrending and bandpass filtering were performed (0.01–0.08 Hz); and nuisance covariates (six head‐motion parameters, global mean signals, white matter signals, and cerebrospinal fluid signals) were regressed out. Finally, for each subject, the mean time course in each seed region was extracted, and Pearson's correlation coefficients between the mean time series of ROIs and the entire brain were computed; these coefficients were converted to *z*‐values using Fisher's r‐to‐z transformation to improve normality. A two‐sample *t* test was used between the rTLE patients and healthy controls. Significance was set at *p* < .05, and AlphaSim correction was performed (single voxel *p* < .01, cluster size > 18). Spearman's rank correlation was performed between the z‐FC values and either BEN values or alertness RTs in rTLE patients (*p* < .05).

## 3 RESULTS

### 3.1 Alertness performance

The intrinsic alertness RT and phasic alertness RT were significantly longer in the rTLE patients than in the healthy controls, suggesting an impairment of the alertness function in the rTLE patients. However, there was no significant difference in the alertness network RTs between the two groups. Additional details are provided in Table 1.

**Table 1:** Attention network test results for patients with rTLE and healthy controls

Alertness performance (ms)	Patients with rTLE (mean ± *SD*)	Controls (mean ± *SD*)	*p*‐value
Alertness network RT	37.21 ± 27.84	44.02 ± 19.01	.254
Intrinsic alertness RT	685.44 ± 111.45*	596.02 ± 54.97	.000
Phasic alertness RT	648.23 ± 115.84*	552.00 ± 54.67	.000

Abbreviations: ms, millisecond; RT, reaction time; *SD*, standard deviation.

* Significance was set at *p* < .05.

### 3.2 BEN alterations in the rTLE patients

**Figure 2:**
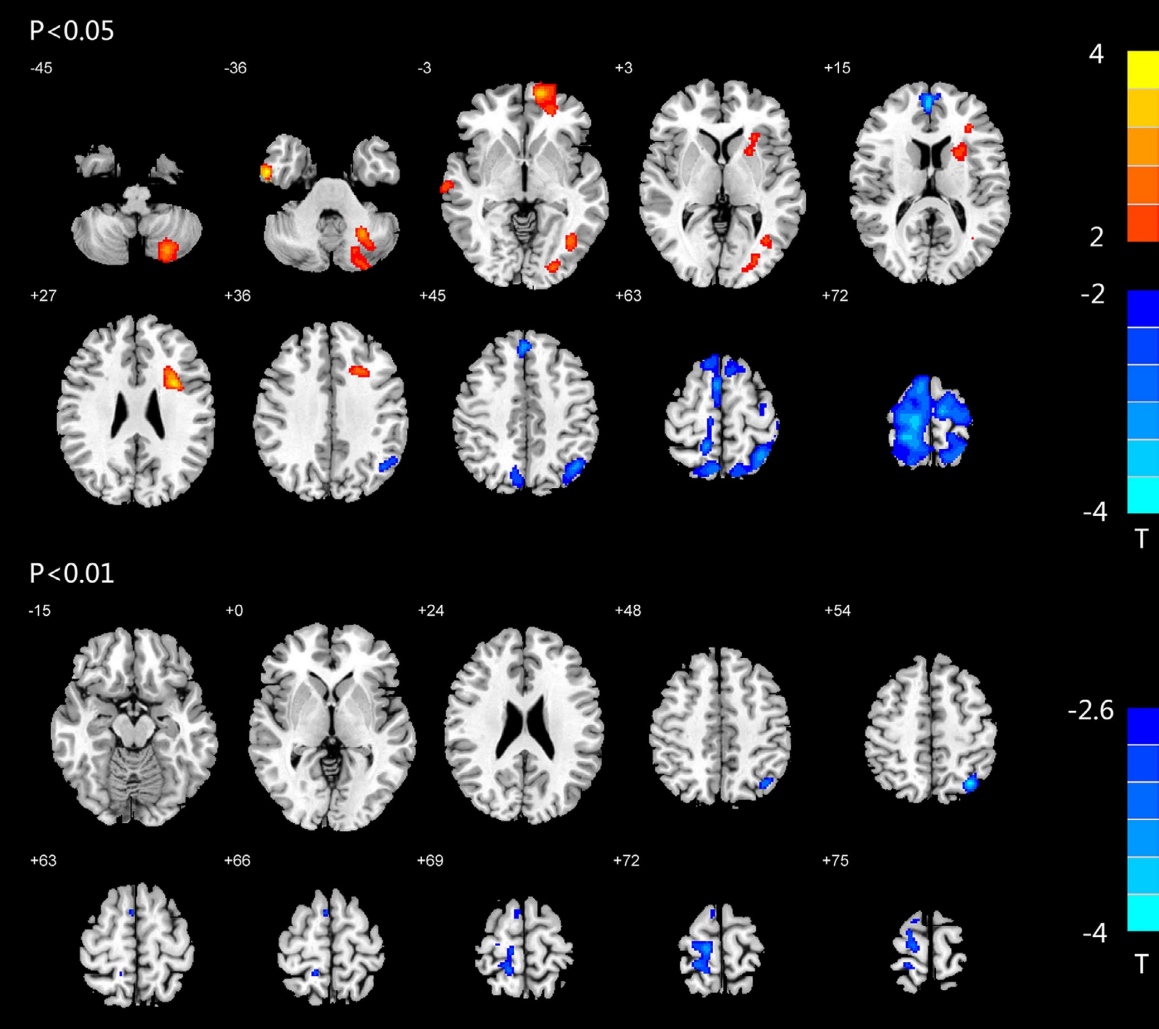
Altered BEN in rTLE patients. BEN differences between rTLE patients and healthy controls. Red and blue colors indicate increased and decreased BEN, respectively. The colored bars indicate the range of *t*‐values; *T*: *t*‐values; *p* < .05, *p* < .01: Statistical threshold of AlphaSim correction, voxel > 140

As shown in Figure 2 and Table 2, significant increases in BEN were observed in the right middle temporal gyrus, inferior temporal gyrus, left superior and middle frontal gyrus, left middle occipital gyrus, left insula, putamen, and left posterior cerebellum; in contrast, BEN decreased significantly in the right middle frontal gyrus and left supplementary motor area (*p* < .05, AlphaSim‐corrected, single voxel *p* < .01, cluster size > 140). At *p* < .01, BEN decreased in patients with rTLE in the left superior parietal lobule and right precentral gyrus; these two brain regions were defined as ROI1 and ROI2, respectively.

**Table 2:** Brain areas with BEN alterations in rTLE patients compared with healthy controls

	Anatomic location	Peak MNI coordinates (*x*, *y*, *z*)	cluster size (voxels)	*t*‐value
*p* < .05, AlphaSim‐corrected, cluster > 140				
rTLE > Control	Left posterior cerebellum	−26, −58, −38	743	3.0186
	Right inferior temporal gyrus	56, −6, −34	308	3.4016
	Left superior and middle frontal gyrus	−14, 60, −2	703	3.1121
	Right middle temporal gyrus	68, −14, −6	168	2.7948
	Left middle occipital gyrus	−24, −88, −2	158	2.5633
		−40, −66, 0	169	2.6479
	Left insula, putamen	−32, 8, 28	990	3.2023
rTLE < Control	Right middle frontal gyrus	2, 54, 16	283	−2.962
	Left supplementary motor area	−34, −66, 52	7,369	−3.2871
*p* < .01, AlphaSim‐corrected, cluster > 140				
rTLE < Control	Left superior parietal lobule (ROI1)	−34, −66, 52	197	−3.2871
	Right precentral gyrus (ROI2)	12, −26, 72	370	−3.0695

Abbreviation: MNI, Montreal Neurological Institute.

### 3.3 Association of alertness with BEN

The significantly different clusters in the BEN maps between the two groups were defined as ROIs. BEN was not found to be associated with the alertness network RT (*p* = .451, *p* = .760). The intrinsic alertness RT and phasic alertness RT exhibited positive correlations with BEN in the right precentral gyrus (ROI2, *r*_s_ = .441, *p* = .013; *r*_s_ = .429, *p* = .016, respectively). No correlation was found between the BEN values of ROI1 and either the intrinsic alert RT (*p* = .315) or the phasic RT (*p* = .494).

### 3.4 rs‐FC alterations in the ROIs of TLE patients

**Figure 3:**
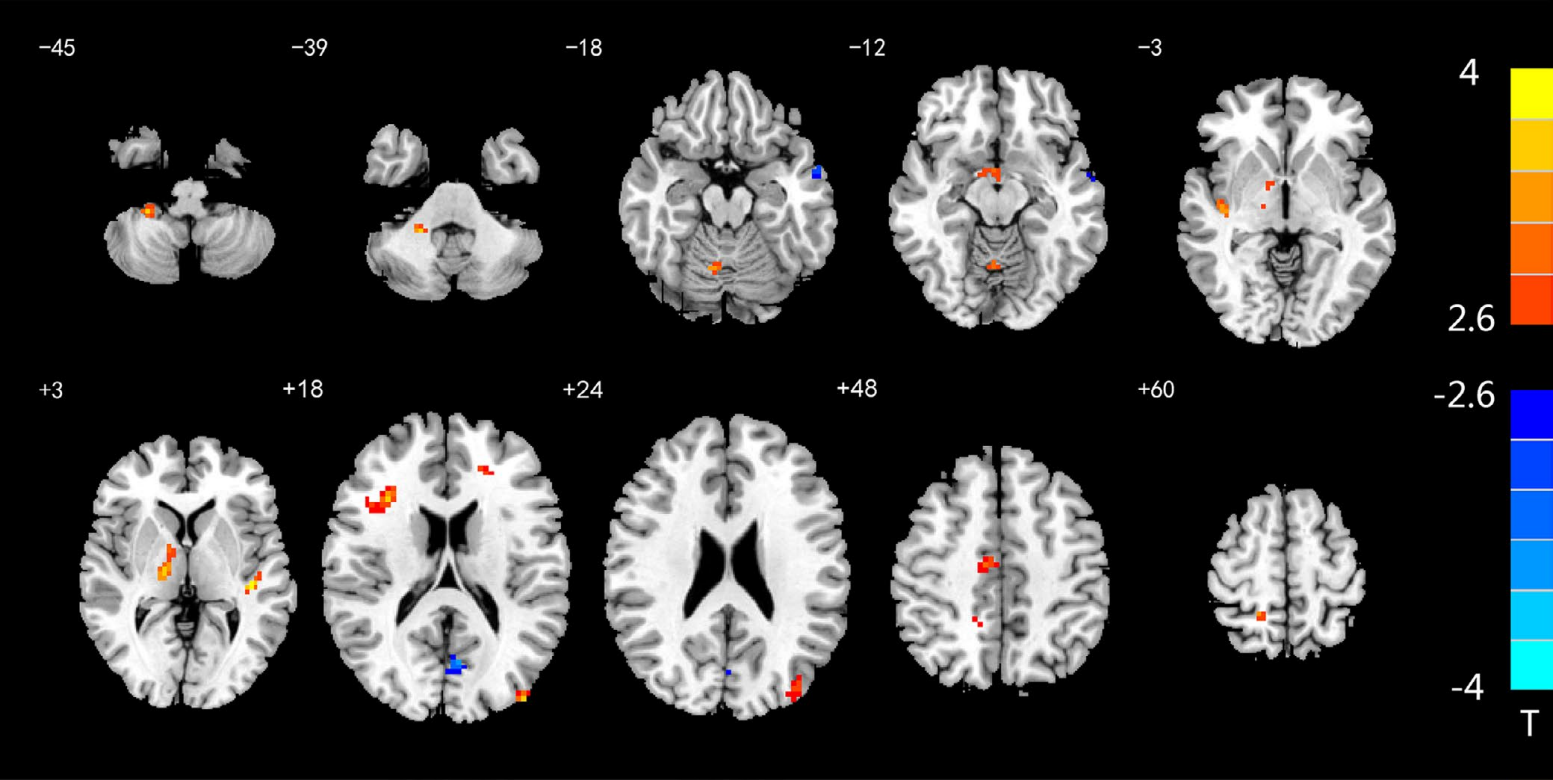
Altered rs‐FC between ROI1 and the whole brain. The rs‐FC differences between rTLE patients and healthy controls in ROI1. Red and blue colors indicate increased and decreased FC, respectively. The colored bars indicate the range of *t*‐values; *T*: *t*‐values; *p* < .01: statistical threshold of AlphaSim correction, voxel > 18

**Figure 4:**
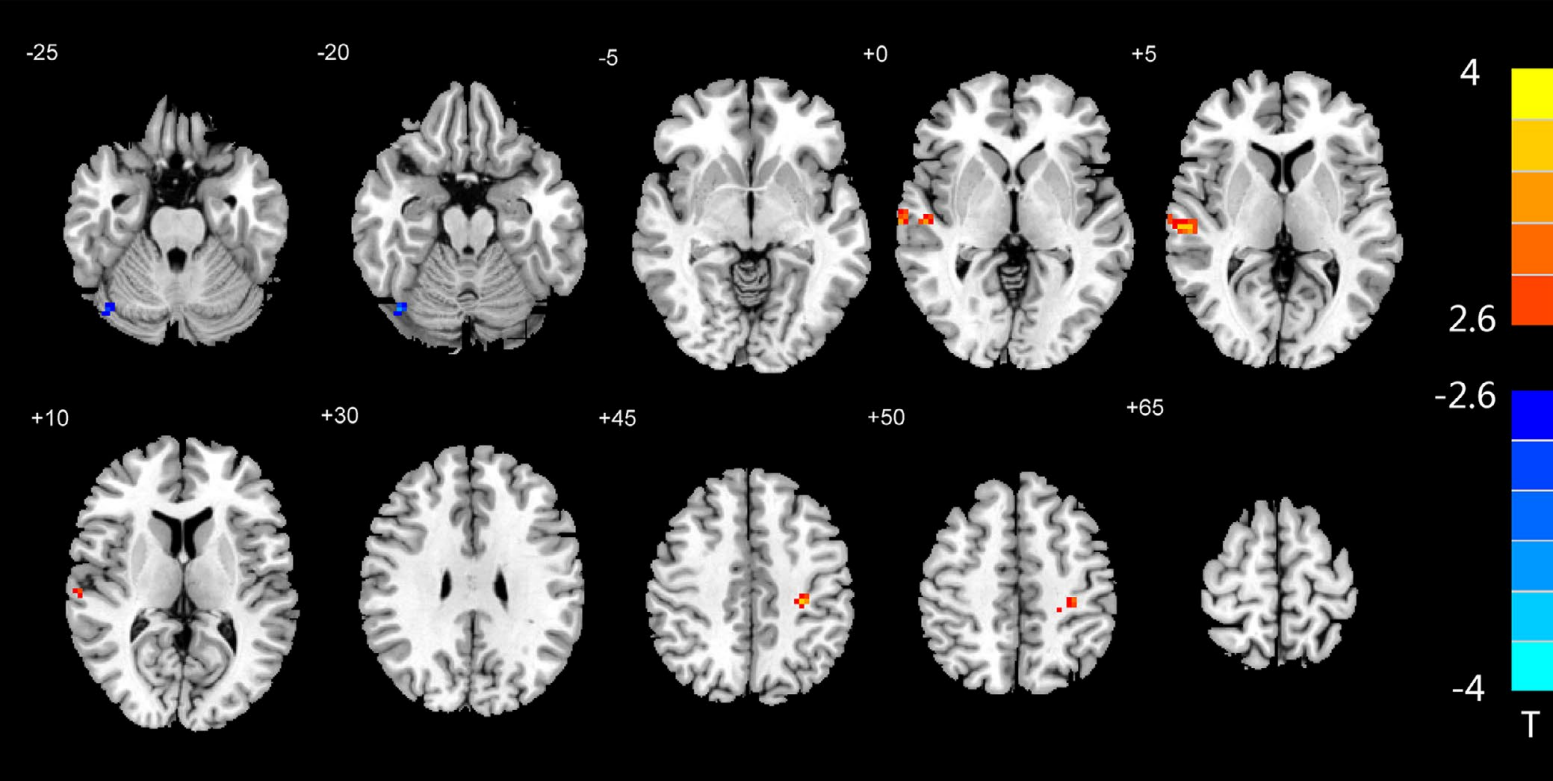
Altered rs‐FC between ROI2 and the whole brain. The rs‐FC differences between rTLE patients and healthy controls in ROI2. Red and blue colors indicate increased and decreased FC, respectively. The colored bars indicate the range of *t*‐values; *T*: *t*‐values; *p* < .01: Statistical threshold of AlphaSim correction, voxel > 18

As shown in Table 3, the functional connectivity between the regions with altered BEN values and the whole brain was calculated, and a two‐sample *t* test was performed (*p* < .01, AlphaSim‐corrected, single voxel <0.01, cluster size >18). We found that, compared with healthy controls, the rs‐FC of ROI1 and the whole brain in patients with rTLE were increased in the posterior lobe, anterior cerebellar lobe, thalamus, insula, middle cingulate gyrus, precuneus, and subcortical inferior frontal gyrus of the right hemisphere and in the subcortical middle frontal gyrus, middle occipital gyrus and superior temporal gyrus of the left hemisphere and was decreased in the left middle temporal gyrus and precuneus (Figure 3). The rs‐FC of ROI2 was increased in the right superior temporal gyrus and left subcortical postcentral gyrus and decreased in the right fusiform gyrus in the rTLE patients compared with the healthy controls (Figure 4).

**Table 3:** Differences in functional connectivity between ROI and the whole brain in patients with rTLE and healthy controls

	Anatomic location	Peak MNI coordinates (*x*, *y*, *z*)	Cluster size (voxel)	*t*‐value
ROI1				
rTLE > Control	Right posterior lobe of the cerebellum	21, −45, −39	23	3.7237
	Right anterior lobe of the cerebellum	9, −63, −18	27	3.4528
	Right thalamus	15, −18, 3	59	3.7751
	Right insula	42, −18, −6	18	3.4779
	Left superior temporal gyrus	−42, −27, 3	19	3.8711
	Right subcortical of the inferior frontal gyrus	33, 21, 18	30	3.7856
	Left subcortical of the middle frontal gyrus	−21, 39, 15	18	3.2845
	Left middle occipital gyrus	−42, −90, 18	26	3.7574
	Right middle cingulate gyrus	0, −12, 42	28	3.3657
	Right precuneus	18, −48, 57	21	3.5933
rTLE < Control	Left precuneus	−6, −69, 18	22	−3.5064
	Left middle temporal gyrus	−60, −3, −15	18	−3.1872
ROI2				
rTLE > Control	Right superior temporal gyrus	54, −18, 3	52	4.1138
	Left subcortical of the postcentral gyrus	−30, −27, 45	18	3.742
rTLE < Control	Right fusiform gyrus	42, −72, −18	18	−3.6977

Abbreviation: MNI, Montreal Neurological Institute.

### 3.5 FC Alertness, FC‐BEN correlations

No significant relationships were found between the alertness RTs and the FC value of the ROIs (*p* = .135–.779). We also did not find a correlation between the average rs‐FC values of the ROIs and the BEN values (*p* = .169 and *p* = .277 for ROI1 and ROI2, respectively).

## 4 DISCUSSION

Cognitive impairment is a widespread phenomenon among TLE patients (Celiker, Yuksel, Tekin, Sariahmetoglu, & Atakli, 2019; Jokeit, Luerding, & Ebner, 2000). According to behavioral research, rTLE patients have significantly lower intrinsic alertness and phasic alertness RTs than healthy individuals. Our results are similar to those of Cerminara et al.'s study (Cerminara et al., 2013) of childhood absence epilepsy as well as those of other previous studies (Chen et al., 2015; Zheng et al., 2012). However, we did not observe a difference in the alertness RT between patients and healthy controls. Four possible explanations exist for this lack of difference: (a) the target signals were present for only a short period of time, and the subjects were therefore susceptible to factors such as distraction and reduced cooperation (Ishigami & Klein, 2011); (b) Zhou, Wang, Wang, Meng, and Fan (2006) have suggested that the type of warning signals in the ANT test and the target state have strong influences on the test results and that there is an interaction between these factors; (c) the alertness RTs of the rTLE patients and healthy controls were either very small, the differences between these two groups could not be detected, or the alertness of the TLE patients was still within the scope of compensation, and, therefore, no differences in alertness were detected compared with the controls (Zheng et al., 2012); and (d) the limited sample size may explain why the alertness RT did not show any differences.

The measurement of BEN by rs‐fMRI is a novel method for assessing brain activity (Wang et al., 2014). To the best of our knowledge, this study represents the first application of rs‐fMRI to study BEN in TLE patients. Unlike other indicators that are measured in rs‐fMRI, such as FC, ALFF, and ReHo, BEN reflects the randomness and irregularity of brain activity and represents the state of the brain's temporal dynamics. Higher entropy indicates a higher degree of randomness and complexity of a system, suggesting that the dynamic system activity is less predictable and organized. This increased entropy may be due to greater vulnerability to internal or external disruptions (Zhou, Zhuang, et al., 2016). In contrast, a reduction in entropy indicates increased regularity and reduced complexity of brain activity. The rTLE patients had both increased and reduced resting BEN compared with that of the normal controls, which suggests both functional impairment and compensation in rTLE patients. This study provides novel information on brain function abnormalities in rTLE patients.

The temporal lobe is commonly viewed as the main region affected in TLE, and the findings of our study that the BEN was increased in the right inferior temporal gyrus and middle temporal gyrus support this viewpoint. Mankinen et al. (2011) reported increased ReHo values in the posterior cingulate gyrus and right temporal lobe of TLE patients and decreased values in the cerebellum. These results are consistent with our findings regarding right temporal lobe damage. Tsuda et al. (2018) found reduced FA and increased MD and RD in widespread white matter regions of patients with TLE and that these changes were related to the duration of illness. As recurrent epileptiform discharges change the structure and function of both the temporal lobe and the entire brain (Maccotta et al., 2013), we found that BEN was significantly increased not only in the right middle temporal gyrus but also in other regions, such as the superior and middle frontal gyri, middle occipital gyrus, insula, putamen, and posterior cerebellum of the left hemisphere; these findings may have resulted from damage caused by epileptic discharges from the right temporal lobe to these brain regions, resulting in increased BEN. Liao et al. (2010) reported that, compared with controls, node strength and functional connectivity significantly increased not only in the temporal gyrus but also in other brain regions in TLE patients. Lieb, Dasheiff, and Engel (2012) assessed temporal lobe epilepsy using electroencephalography and suggested that the epileptic focal discharge primarily spreads to the frontal lobes. Regions with increased BEN are mainly related to memory functions (temporal lobe), the executive control network (middle frontal gyrus), somatovisceral sensory processing (insula), alertness functions (putamen), visual networks (occipital gyrus), and language functions (posterior lobe) (Hu et al., 2012). It is well known that multiple brain dysfunction is caused by the frequent synchronous discharge of neurons in epileptic patients. As a result, memory (McDonald et al., 2008), alertness (Zheng et al., 2012), the executive control network (Dabbs, Jones, Seidenberg, & Hermann, 2009; Oyegbile et al., 2018), and language functions (Trimmel et al., 2018) suffer varying degrees of damage. Moreover, seizures in patients with TLE are often foreshadowed by abdominal sensations of rising air or visual hallucinations; these sensations are abnormalities of the somatovisceral and visual networks. Recurrent abnormal discharges of neurons in the brain are caused by increases in the irregularity of brain activity. Jouny CC's (Jouny & Bergey, 2012) EEG study showed that sample entropy increased at the early onset of partial seizures and in mesial onset seizures in epileptic patients. These results are in accordance with Vaillancourt and Newell's hypothesis (Vaillancourt & Newell, 2002), which states that brain complexity is increased in individuals with disease and in elderly individuals. However, since the mechanism has not yet been reported, this area still needs further research.

Brain entropy was significantly reduced in the right middle frontal gyrus and left supplementary motor area (*p* < .05, AlphaSim‐corrected, single voxel *p* < .01, voxel > 140). At *p* < .01, BEN decreased in the left superior parietal lobule and right precentral gyrus of patients with rTLE, suggesting increased regularity of brain activity in these regions. Entropy reductions in relevant brain regions may function as compensatory mechanisms (Zhou, Zhuang, et al., 2016). The patients in this study may be in an early stage of TLE; through the reconstruction of the brain functional connectivity, it can be compensated to a certain extent. Our correlation analysis of BEN alterations and behavior supports this finding. Although brain entropy was not correlated with the alertness network RT, the BEN value in the ROI2 (right precentral gyrus) was positively correlated with the intrinsic and phasic alertness RTs. Lower entropy values were associated with shorter RTs, which indicated better alertness functions. Notably, entropy was decreased in the right precentral gyrus as a form of compensation to preserve brain function. The right precentral gyrus is a component of the motion center of the brain and has an influence on alertness function. Sturm et al. (2004) used positron emission computed tomography (PET) to show that in a task of intrinsic alertness (auditory stimulation), the regional cerebral blood flow (rCBF) increased in several regions of the brain in healthy individuals, including the right precentral gyrus, anterior cingulate gyrus, and other brain regions. Those authors stressed that the right precentral gyrus plays an important role in intrinsic alertness. In a study of patients with ADHD, Cao et al. (2008) found that activation is reduced in the right precentral gyrus, right supplementary motor area and left putamen during alertness tasks compared with that in healthy controls. We suggest that the BEN alterations in the right precentral gyrus may be correlated with changes in phasic and intrinsic alertness for compensatory purposes. Among the many functions of the precentral gyrus, the most important is motor function. Therefore, we analyzed the correlation between the execution time (RT = 105.96 + 35.69 ms) of the patients in the ANT test and the BEN of the two ROIs, and we found no significant correlation (*p*1 = .077, *p*2 = .161 for ROI1 and ROI2, respectively). The Attention Network theory proposed by Posner (2008) holds that the attention function consists of three submodules: alert, directional, and executive functions. Notice that the network is not responsible for a single brain area; alertness, orientation, and execution are relatively independent and interdependent, constituting the neural network connection within the attention network and cooperating with each other to complete the attention function. Therefore, we cannot fully guarantee that entropy is only related to alertness or motor response preparation or planning. This needs to be confirmed by further studies.

In the analysis of functional connectivity, we calculated the rs‐FC of the two brain regions with altered BEN, which were considered ROIs. The FC between the ROIs and the whole brain showed an increasing trend in rTLE patients compared with healthy controls. Most of these brain regions where FC increased, such as the thalamus, right precuneus, and middle frontal gyrus, are involved in the default mode network (DMN), whereas the two brain regions (left middle temporal gyrus and left precuneus) where FC decreased are also involved in the DMN. Li, Fang, Hager, Rao, and Wang (2016) found that compared with controls, chronic smokers have a globally higher BEN and that lower entropy in the default networks was associated with more years of smoking. They suggested that the damage caused by severe nicotine dependence requires stronger regular activity to compensate for it. In a seed‐based analysis of DMN, Haneef, Lenartowicz, Yeh, Engel, and Stern (2012, 2014) used rs‐fMRI to show that connectivity was reduced between the anterior and posterior regions involved in the DMN in patients with TLE. Moreover, FC between the DMN and other brain areas was increased in patients with left TLE and in patients with right TLE. In a study based on MEG, Hsiao et al. (2015) found that the resting‐state functional connectivity of the default network in patients with TLE was significantly higher than that in healthy subjects. In general, a decrease in FC in brain areas that show increased entropy occurs because the brain experiences difficulty in establishing a phasic corresponding relationship; in contrast, low entropy in a brain area will enhance functional connectivity of that region (Zhou, Huang, et al., 2016). In our study, there were both increases and decreases in rs‐FC between regions with reduced BEN and other brain areas, with a tendency toward increased rs‐FC. This phenomenon may indicate the process of brain functional reconstruction, as some brain areas were damaged by epileptic discharge, whereas other brain regions appeared to compensate. It is a simultaneous process. The tendency toward functional connectivity in the brain of TLE patients will be increased or decreased, which may be related to the severity and course of the disease. This explanation is consistent with the results of a study by Zhou, Huang, et al. (2016), who found that changes in the functional connectivity of a region are related to the brain areas with altered entropy, and there is an association between the altered rsFC and clinical variables of sleep quality in patients with chronic primary insomnia.

We did not find significant correlations between the FC values of the ROIs and the corresponding BEN values, possibly due to the small number of subjects or differences in disease duration or severity, all of which can lead to inconsistent observed degrees of compensation. The reconstruction of brain functional connections may be asynchronous. In addition, brain entropy based on fMRI can be used as an index to evaluate the complexity of a time series, while functional connectivity is used to calculate the Pearson correlation coefficients of two time series and characterize their synchronization or statistical dependence. The information presented by the two methods is different, and their correlation may not be revealed by simple rank correlation or linear correlation analysis.

In this study, there were several limitations. We enrolled patients with rTLE but not those with left temporal lobe epilepsy (lTLE). Whether BEN is altered in patients with lTLE is unknown; this question will be investigated in a future study. The current study did not include other classical analyses of rs‐MRI, such as ReHo or ALFF analyses. In the future, these parameters can be simultaneously analyzed and compared with BEN. In addition, the study did not group patients according to the use of different medications or duration of medication; thus, we do not know whether the medication choice or duration has an effect on brain entropy in patients. Finally, the fMRI scans were performed during the interictal period of the subjects, and the direct effects of seizures on brain entropy are not yet clear. This is another aspect of future research.

Finally, as the brain is one of the most complex organs, the understanding and recognition of its functions and compensatory patterns require more research. We suggest that BEN mapping may provide a useful tool for probing brain mechanisms related to TLE.

## CONFLICT OF INTEREST

None declared.

## Data Availability

The data that support the findings of this study are available from the corresponding author upon reasonable request.
